# Chromatin as dynamic 10-nm fibers

**DOI:** 10.1007/s00412-014-0460-2

**Published:** 2014-04-16

**Authors:** Kazuhiro Maeshima, Ryosuke Imai, Sachiko Tamura, Tadasu Nozaki

**Affiliations:** 1 Biological Macromolecules Laboratory, Structural Biology Center, National Institute of Genetics, Mishima, Shizuoka 411-8540 Japan; 2 Department of Genetics, School of Life Science, Graduate University for Advanced Studies (Sokendai), Mishima, Shizuoka 411-8540 Japan; 3 Institute for Advanced Biosciences, Keio University, Fujisawa, 252-8520 Japan

## Abstract

Since Flemming described a nuclear substance in the nineteenth century and named it “chromatin,” this substance has fascinated biologists. What is the structure of chromatin? DNA is wrapped around core histones, forming a nucleosome fiber (10-nm fiber). This fiber has long been assumed to fold into a 30-nm chromatin fiber and subsequently into helically folded larger fibers or radial loops. However, several recent studies, including our cryo-EM and X-ray scattering analyses, demonstrated that chromatin is composed of irregularly folded 10-nm fibers, without 30-nm chromatin fibers, in interphase chromatin and mitotic chromosomes. This irregular folding implies a chromatin state that is physically less constrained, which could be more dynamic compared with classical regular helical folding structures. Consistent with this, recently, we uncovered by single nucleosome imaging large nucleosome fluctuations in living mammalian cells (∼50 nm/30 ms). Subsequent computational modeling suggested that nucleosome fluctuation increases chromatin accessibility, which is advantageous for many “target searching” biological processes such as transcriptional regulation. Therefore, this review provides a novel view on chromatin structure in which chromatin consists of dynamic and disordered 10-nm fibers.

## Introduction

There are 60 trillion cells in the human body. Each cell contains 2 m of genomic DNA in a small nucleus with an approximately 10-μm diameter (a volume of only ∼100 fL to 1 pL), and yet, it is able to search and read the information in its genomic DNA to execute diverse cellular functions. Therefore, it is important to understand how this long genomic DNA is organized in the nucleus. In the nineteenth century, W. Flemming described a nuclear substance that was clearly visible after staining with a basic dye using primitive light microscopes and named it “chromatin.” This is now thought to be the basic unit of genomic DNA organization (Olins and Olins 2003). Since then, even before the discovery of the structure of DNA (Watson and Crick 1953), chromatin has attracted significant interest from biologists. In this review article, we assess the available data to provide a novel view of chromatin in which “chromatin is a dynamic and disordered 10-nm fiber.”

## DNA and nucleosomes

**Fig. 1:**
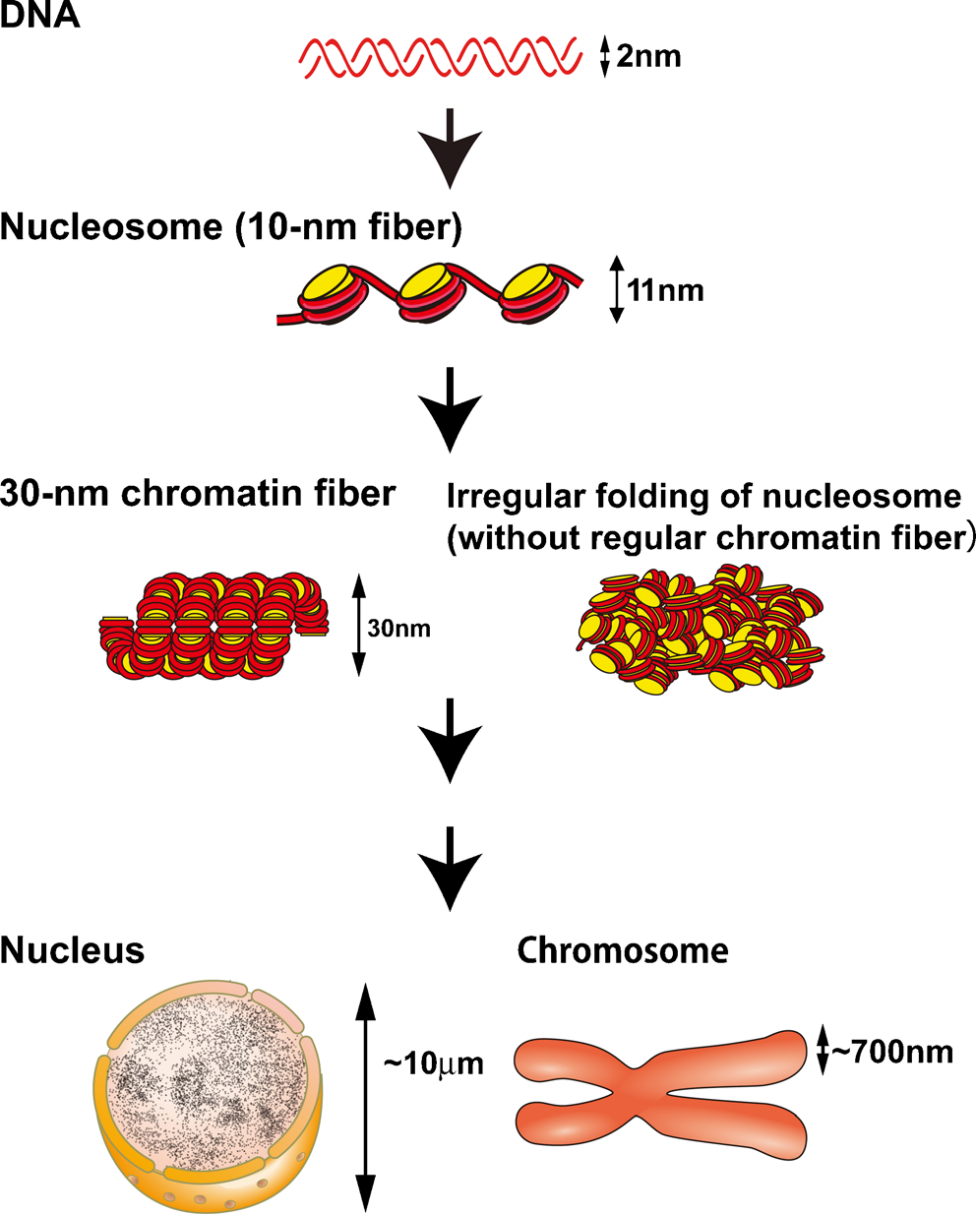
Old and novel views of chromatin structure. A long DNA molecule with a diameter of ∼2 nm is wrapped around a core histone octamer and forms a nucleosome with a diameter of 11 nm (Alberts et al. 2007). The nucleosome has long been assumed to fold into 30-nm chromatin fibers (*left*) and subsequently into the higher order organization of interphase nuclei or mitotic chromosomes. The *right panel* shows the novel hypothesis of irregularly folded nucleosome fibers

Deoxyribonucleic acid (DNA) is a negatively charged polymer that produces electrostatic repulsion between adjacent DNA regions. Therefore, it would be difficult for a long DNA molecule alone to fold into a small space like the nucleus (Bloomfield 1996; Yoshikawa and Yoshikawa 2002). To overcome this problem, the long, negatively charged polymer is wrapped around a basic protein complex known as a core histone octamer, which consists of the histone proteins H2A, H2B, H3, and H4, to form a nucleosome (Fig. 1) (Olins and Olins 1974; Kornberg 1974; Woodcock et al. 1976). The structure of a nucleosome is well known at atomic resolution (1.9 Å) (Davey et al. 2002): 147 base pairs (bp) of DNA are wrapped in 1.7 left-handed superhelical turns around a histone octamer, whose surface is positively charged. Each nucleosome particle is connected by linker DNA (20–80 bp) to form repetitive motifs of ∼200 bp; this was described originally to resemble “beads on a string” (Fig. 1) (Olins and Olins 2003). This nucleosome fiber is also known as the 10-nm fiber (Fig. 1). A single histone octamer in the nucleosome has ∼220 positively charged lysine and arginine residues and ∼74 negatively charged aspartic acid and glutamic acid residues. There are also 400 negative charges in the phosphate backbone of 200 bp of DNA. Because only about half of the negative charges in the DNA are neutralized, the remaining charge must be neutralized by other factors (e.g., linker histone H1, cations, and other positively charged molecules) for further folding.

## Discovery of 30-nm chromatin fibers in vitro

**Fig. 2:**
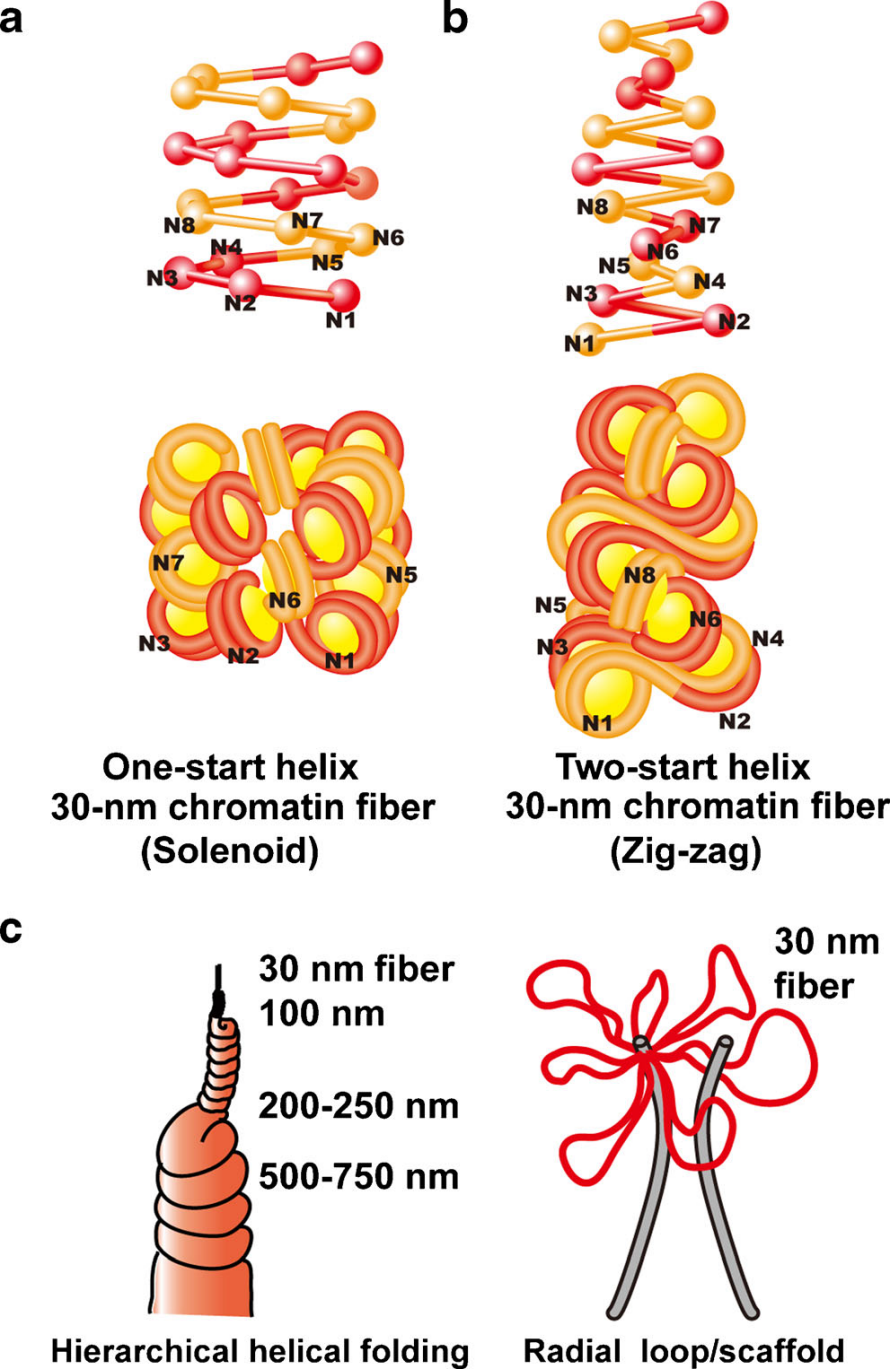
Two classical models of 30-nm chromatin fibers and higher order chromatin structures. **a** One-start helix (solenoid), **b** two-start helix (zigzag). (*Top*) A scheme of the two different topologies of chromatin fibers is shown (Robinson and Rhodes 2006). Positions from the first (*N1*) to the eighth (*N8*) nucleosome are labeled. **c** Two classical higher order chromatin structure models: the hierarchical helical folding model (Sedat and Manuelidis 1978) and the radial loop model (Laemmli et al. 1978). In the radial loop model, many loop structures of the 30-nm fiber (*red*) wrap around the scaffold structure (*gray*) (Laemmli et al. 1978), which consists of condensin and topoisomerase IIα (Maeshima and Laemmli 2003)

In 1976, Finch and Klug first found, under transmission electron microscopy (EM), that purified nucleosome fibers (10-nm fibers) with linker histone H1 or Mg^2+^ ions were folded into fibers with a diameter of 30 nm. They named these fibers “30-nm chromatin fibers” (Figs. 1 and 2a, b) (Finch and Klug 1976). In their structural model of the 30-nm fibers called “solenoids,” consecutive nucleosomes are located adjacent to one another in the fiber and folded into a simple “one-start helix” (Fig. 2a). Subsequently, a second model of the “two-start helix” was proposed based on microscopic observations of isolated nucleosomes (Fig. 2b) (Woodcock et al. 1984). The second model assumed that nucleosomes were arranged in a zigzag manner, where a nucleosome in the fiber was bound to the second neighbor (Bassett et al. 2009) (Fig. 2b). In addition to these two famous structural models, many other structural variations of 30-nm chromatin fibers have been proposed (van Holde and Zlatanova 2007).

Although 30-nm chromatin fibers have been studied extensively using various techniques, including biochemistry, biophysics, X-ray crystallography, conventional EM, cryo-EM, and small-angle X-ray scattering (SAXS) (Finch and Klug 1976; Woodcock et al. 1984; Widom and Klug 1985; Dorigo et al. 2004; Schalch et al. 2005; Robinson et al. 2006; Bordas et al. 1986; Langmore and Paulson 1983; Hansen 2002; Gilbert et al. 2004; Bystricky et al. 2004; Kruithof et al. 2009), their definitive structure remains elusive (van Holde and Zlatanova 2007; Tremethick 2007; for more recent reviews, see Grigoryev and Woodcock 2012; Ghirlando and Felsenfeld 2013; Bian and Belmont 2012). Defining a specific structure for the 30-nm chromatin fibers may not be valid for several reasons. First, the Rhodes group suggested that the solenoid or zigzag method of compaction was defined by the length of the nucleosomal linker DNA (Routh et al. 2008). Second, Grigoryev et al. demonstrated that two-start zigzag and one-start solenoids could be present simultaneously in a 30-nm chromatin fiber under certain conditions (Grigoryev et al. 2009; Grigoryev and Woodcock 2012). It should be emphasized that even a variation in linker DNA length of 1 bp will correspond to a 36° rotation of one nucleosome with respect to its neighbor and will cause significant structural changes in the fiber (van Holde and Zlatanova 2007).

Although the defined structure of 30-nm chromatin fibers remains unclear, it has long been assumed that the 10-nm nucleosome fibers form a 30-nm chromatin fiber and, subsequently, the higher order chromatin structures of interphase nuclei and mitotic chromosomes. Several models have been proposed to describe the structure of higher order chromatin. The “hierarchical helical folding model” suggests that a 30-nm chromatin fiber is folded progressively into larger fibers, including ∼100-nm and then ∼200-nm fibers, to form large interphase chromatin fibers (chromonema fibers) or mitotic chromosomes (Fig. 2c) (Sedat and Manuelidis 1978; Belmont et al. 1989; Belmont and Bruce 1994; for a review, see Horn and Peterson 2002). In contrast, the “radial loop model” assumes that a 30-nm chromatin fiber folds into radially oriented loops to form mitotic chromosomes (Fig. 2c) (Paulson and Laemmli 1977; Laemmli et al. 1978; Marsden and Laemmli 1979).

## Does the 30-nm chromatin fiber exist in vivo? The cryo-EM study

In 1986, the Dubochet group performed a pioneering study to visualize native cellular structures using cryo-EM (Dubochet et al. 1986). Mammalian mitotic cells were frozen rapidly, sectioned, and observed directly under a cryo-EM with no chemical fixation or staining (cryo-EM of vitreous sections, CEMOVIS). The Dubochet group first observed “native” mammalian chromosomes in these sections. Mitotic chromosomal regions were apparent because they were excluded from electron-dense ribosomes and, therefore, were distinguishable from the cytoplasmic regions, which are full of ribosomes (Dubochet et al. 1986; see also Maeshima and Eltsov 2008). Surprisingly, the chromosomes had a homogeneous, grainy texture with ∼11-nm spacing. No higher order or periodic structures, including 30-nm fibers, were observed. This suggested that the basic structure of the chromosome was a liquid-like compact aggregation of 10-nm, not 30-nm, nucleosome fibers (Dubochet et al. 1988).

Interphase chromatin has also been visualized using cryo-EM. Although the chromatin regions in interphase nuclei are not as obvious as those in mitotic chromosomes because there is no efficient chromatin marker in interphase nuclei, it was suggested that interphase nuclei in most higher eukaryote cells might not contain 30-nm chromatin fibers (Dubochet and Sartori Blanc 2001; Bouchet-Marquis et al. 2006; Fakan and van Driel 2007). For example, typical heterochromatin regions in plant or mammalian nuclei resembled mitotic chromosomes by cryo-EM, forming a homogeneous texture without 30-nm structures (Bouchet-Marquis et al. 2006; Fakan and van Driel 2007).

On the other hand, it is unclear whether the absence of 30-nm structures in cryo-EM images truly demonstrates a lack of 30-nm chromatin fibers because when researchers capture cryo-EM images, they use a technique called “defocusing” to produce high-contrast images. This process results in artificial amplification or suppression of the signal intensity, which affects different structural features depending on the defocus value (contrast transfer function [CTF] effect; for a review, see Frank 2006). It is thus possible that the degree of defocusing needed to image chromosomes or chromatin with high contrast prevents the visualization of 30-nm chromatin fibers. To solve this problem, we collaborated with Eltsov, Frangakis, and Dubochet to compensate for the CTF effect by merging several images taken at different levels of defocus into a single image (Conway and Steven 1999). Even after this correction, we were unable to detect 30-nm structures in the chromosomal areas. In addition, the detection of periodic structures in the chromosomal region by power spectral (Fourier transform) analysis revealed a prominent peak at 11 nm, but not at 30 nm. This cryo-EM study suggested that 30-nm chromatin fibers were essentially absent from mitotic chromosomes; therefore, we proposed that 10-nm nucleosome fibers exist in a highly disordered, interdigitated state similar to a “polymer melt” (Figs. 1 and 4) (Eltsov et al. 2008; Maeshima et al. 2010a).

## Small-angle X-ray scattering analyses revealed no 30-nm chromatin structures in interphase nuclei and mitotic chromosomes

**Fig. 3:**
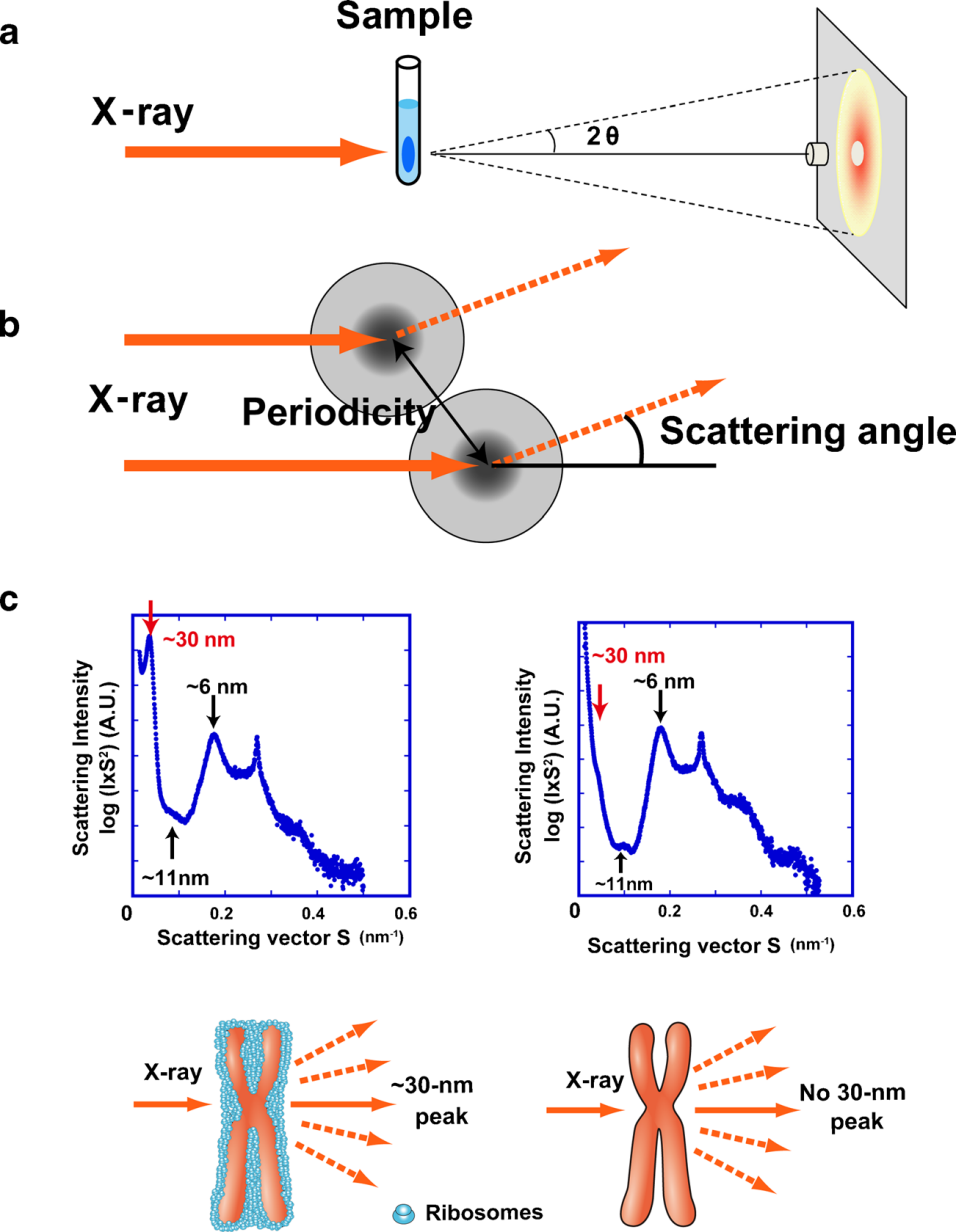
Small angle X-ray scattering (SAXS) analysis of chromatin structure. **a** Experimental design. The chromosome pellet in a quartz capillary tube was exposed to synchrotron X-ray beams, and the scattering patterns were recorded using the imaging plate (Nishino et al. 2012). **b** When non-crystal materials were irradiated with X-rays, scattering at small angles generally reflected periodic structures. Images **a** and **b** were reproduced from Joti et al. (2012), with some modifications. **c** *Upper left* Typical SAXS patterns of purified mitotic HeLa chromosome fractions. Three peaks at ∼6, ∼11 (weak), and ∼30 nm were detected (*arrows*). (*Upper right*) After the removal of ribosome aggregates, the 30-nm peak disappeared, whereas the other peaks remained. (*Bottom*) A model whereby the 30-nm peak in SAXS results from regularly spaced ribosome aggregates and not from the chromosomes. Image **c** was reproduced from Nishino et al. (2012), with some modification

Although our cryo-EM study did not detect any 30-nm structures in mitotic chromosomes, it might be impossible to observe potential hierarchical regular structures because only a small number of 50-nm sections were examined (Eltsov et al. 2008). Langmore and Paulson (Langmore and Paulson 1983; Paulson and Langmore 1983) detected a 30-nm structure in interphase nuclei and mitotic chromosomes using small-angle X-ray scattering (SAXS) analysis, which can detect bulky periodic structures in non-crystal materials in solution without chemical fixation or staining (Fig. 3a, b) (Roe 2000). Therefore, this study provided evidence for the existence of 30-nm chromatin fibers in interphase chromatin and mitotic chromosomes (Langmore and Paulson 1983; Paulson and Langmore 1983). Because these findings were inconsistent with the cryo-EM findings described above, we performed a comprehensive investigation of the structure of interphase nuclei and mitotic chromosomes using SAXS and cryo-EM (Nishino et al. 2012; Joti et al. 2012; for a review, see Hansen 2012). Isolated human interphase nuclei and mitotic chromosomes were exposed to synchrotron X-ray beams (Fig. 3a). A typical scattering pattern of interphase nuclei and mitotic chromosomes exhibited three peaks at 30-, weakly at 11-, and 6-nm (Fig. 3c, left) (Nishino et al. 2012; Joti et al. 2012). This was consistent with the previous findings of Langmore and Paulson (1983), who suggested that the 6- and 11-nm peaks were derived from the face-to-face and edge-to-edge positioning of nucleosomes, respectively. They concluded that the 30-nm peak represented the side-by-side packaging of 30-nm chromatin fibers. However, this fails to explain why the 30-nm structures were not observed in interphase chromatin and mitotic chromosomes using cryo-EM.

To understand the nature of the 30-nm peak observed using SAXS, isolated chromosomes were examined using cryo-EM (Nishino et al. 2012; Joti et al. 2012). Again, no 30-nm chromatin fibers were observed in chromosomes. However, the cryo-EM images revealed that the surface of the chromosome was coated with electron-dense granules the size of ribosomes. Subsequent immunostaining and Western blotting confirmed that the chromosome surface was contaminated with ribosomes. The ribosomes were stacked regularly at ∼30-nm intervals, which could explain the ∼30-nm peak observed using SAXS. To test this hypothesis, we removed ribosomes from the surface of the chromosome by washing with an isotonic buffer-containing polyamine and EDTA (Lewis and Laemmli 1982) while maintaining the size and shape of the chromosomes and then analyzed mitotic chromosomes using SAXS. Importantly, no 30-nm peaks were detected (Fig. 3c, right), but the 11- and 6-nm peaks resulting from the internal structure of the nucleosomes remained (Fig. 3c, right). Similarly, when we examined the nuclei after ribosome removal, the 30-nm peak in the SAXS pattern disappeared (Joti et al. 2012). These results suggested the absence of a 30-nm chromatin fiber in interphase chromatin and mitotic chromosomes.

Next, we investigated the larger scale chromatin structure of interphase nuclei and mitotic chromosomes using a newly developed apparatus for ultra-small-angle X-ray scattering (USAXS) (Nishino et al. 2009). Consistent with our previous observations, there were no regular periodic structures between ∼30- and 1,000-nm in interphase nuclei and mitotic chromosomes. This contradicts the hierarchical helical folding model (Fig. 2c) (Nishino et al. 2012; Joti et al. 2012). The scattering properties also suggested the existence of a scale-free structure or fractal nature up to ∼275-nm in interphase chromatin and ∼1,000-nm in mitotic chromosomes. This suggests that interphase and mitotic chromatin share the common structural features of up to ∼275 nm of condensed and irregularly folded 10-nm nucleosome fibers without 30-nm structures (discussed below). Taken together, the cryo-EM, SAXS, and USAXS data suggest that irregularly folded 10-nm nucleosome fibers form the bulk structure of human interphase chromatin and mitotic chromosomes (Nishino et al. 2012; Joti et al. 2012). Nevertheless, it is possible that short stretches of 30-nm fibers or other regularly folded hierarchies occur in human interphase chromatin and mitotic chromosomes.

## Other evidence supporting the absence of 30-nm chromatin fibers

Dekker (2008) used the chromosome-conformation-capture (3C) technique to investigate the folding of a specific genomic DNA region within yeast cells. He measured the average distance between two loci in the genome by confocal microscopy and the flexibility of the intervening chromatin fiber by the 3C technique. In combination with polymer modeling, the mass density of the chromatin fiber was determined. His conclusion was that yeast chromatin in a transcriptionally active domain did not form a compact 30-nm chromatin fiber but rather was extended with a loose arrangement of 10-nm nucleosome fibers.

More recently, Bazett-Jones et al. used electron spectroscopic imaging (ESI), a process that involves electron microscopy with an energy filter. ESI makes it possible to perform phosphorus and nitrogen mapping in cells with high contrast and resolution (Ahmed et al. 2009; Fussner et al. 2011a, b). The signals from phosphorus and nitrogen, which are the main components of DNA, may be used to assess the folding of genomic DNA and can distinguish 10- from 30-nm fibers. They observed that pluripotent cells were characterized by a highly dispersed mesh of 10-nm, but not 30-nm, fibers (Fussner et al. 2011a, b, 2012). In contrast, differentiated cells form compact chromatin domains leave a large space in the nucleus that is devoid of DNA. Surprisingly, ESI combined with tomography methods revealed that condensed heterochromatin domains such as chromocenters consisted of 10-nm, rather than 30-nm, chromatin fibers (Fussner et al. 2012; for a review, see Quenet et al. 2012), consistent with the observations using cryo-EM. Furthermore, Gan et al. investigated the picoplankton *Ostreococcus tauri*, the smallest known free-living eukaryote, using cryo-EM tomography of ice sections and subsequent computational analysis (Gan et al. 2013). They demonstrated that *O. tauri* chromatin resembles a disordered assembly of nucleosomes without the 30-nm chromatin structure compatible with the polymer melt model. Therefore, several lines of evidence suggest the absence of regular 30-nm chromatin fibers in eukaryotic cells.

The absence of a 30-nm chromatin fiber in native chromatin may not be a surprise. Generally, native chromatin does not have regularly spaced nucleosomes, so linker DNA lengths vary. As pointed out by van Holde and Zlatanova (2007), even the addition of a single base to linker DNA changes the relative orientation of one nucleosome to the next by 36°. Unless nucleosome-nucleosome interactions are sufficient to overcome such variations, the formation of a regular chromatin fiber is impossible.

## Why can 30-nm chromatin fibers be observed in vitro?

**Fig. 4:**
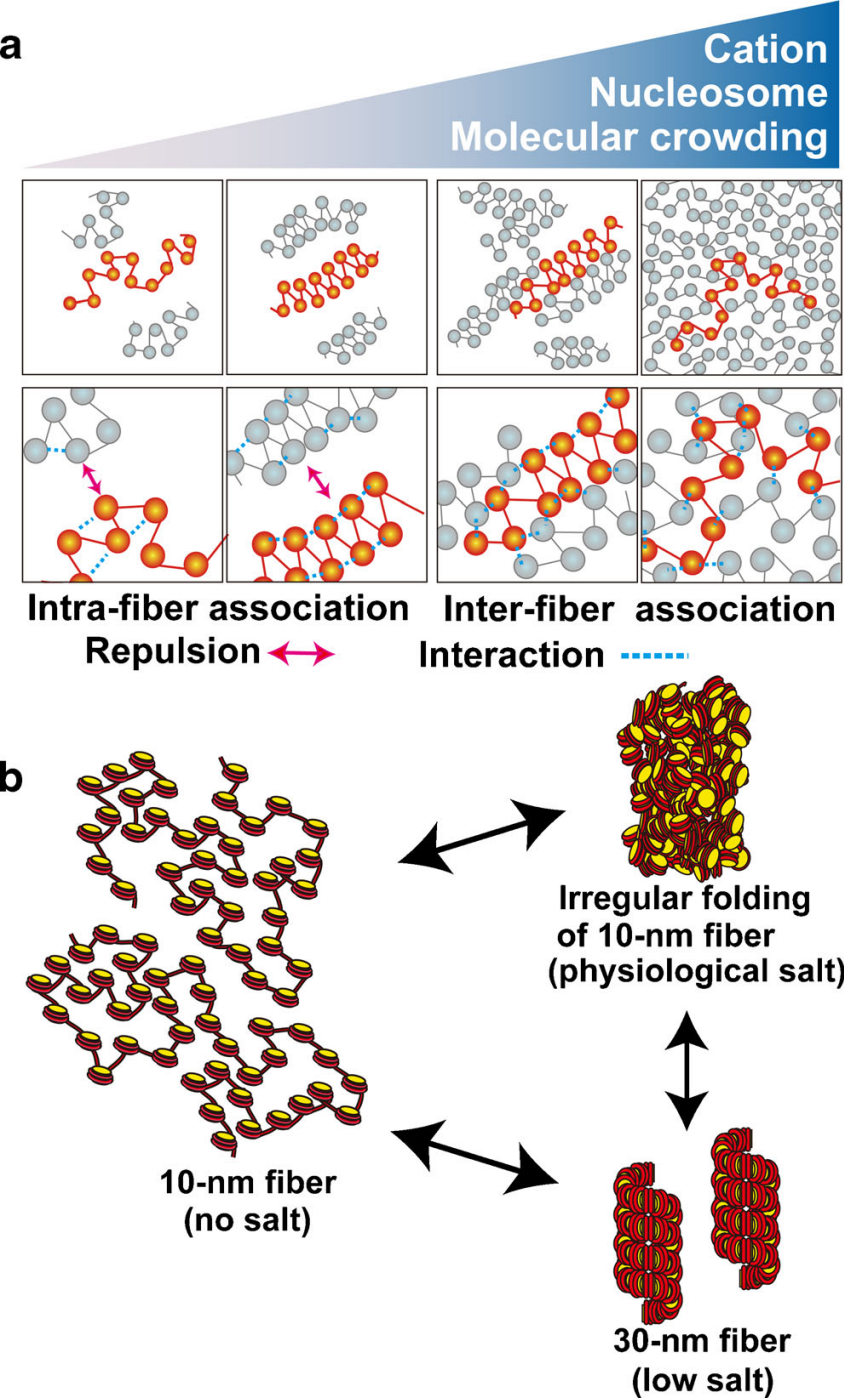
Polymer melt model. **a** Under low-salt conditions, nucleosome fibers could form 30-nm chromatin fibers via intra-fiber nucleosome associations. An increase in salt (cation) concentration results in inter-fiber nucleosomal contacts that interfere with intra-fiber nucleosomal associations, leading to a polymer melt scenario. Note that in these illustrations, we show a highly simplified two-dimensional nucleosome model. *Arrows and dotted lines* show repulsion forces and interactions, respectively. **b** During the melting process, the 30-nm chromatin fibers become irregularly folded nucleosome fibers

Although the near absence of 30-nm chromatin fibers in eukaryotic cells was suggested, these structures are shown in EM images in molecular biology textbooks. We propose that most 30-nm chromatin fibers in EM images are in vitro artifacts caused by the low-salt buffer conditions. The formation of 30-nm chromatin fibers requires the selective binding of nucleosomes, which are close neighbors on the DNA strand, via intra-fiber nucleosomal association (Fig. 4a). In low-salt buffer conditions of <1 mM MgCl_2_ or <100 mM NaCl, nucleosomal fibers gently repel each other due to their negative charges. This “isolation of nucleosome fibers” facilitates the intra-fiber nucleosomal association and the subsequent formation of stable 30-nm chromatin fibers (Fig. 4a, b). In conventional EM imaging studies, these 30-nm fibers might be stabilized through chemical cross-linking (such as glutaraldehyde fixation) and then shrunk further after alcohol dehydration during sample preparation (Maeshima et al. 2010b).

## Polymer melt

It is important to assess chromatin structure under more physiological salt conditions. Under these conditions, inter-fiber nucleosome interactions become increasingly dominant (Fig. 4a, b) (Maeshima et al. 2010a). Nucleosome fibers (10 nm) are forced to interdigitate, which interferes with the formation and maintenance of 30-nm chromatin fibers. This leads to the polymer melt (Maeshima et al. 2010a) or “self-oligomer” state (for a review, see Hansen 2002; Hansen 2012) (Fig. 4a, b). In addition, inter-fiber nucleosome interactions increase significantly in the presence of >2 mM Mg^2+^ ions (Zheng et al. 2005; Kan et al. 2009). However, it is important to note that the tail domain of histone H4 mediates both 30 nm fiber formation (Dorigo et al. 2003) and inter-fiber nucleosome association (Kan et al. 2009). Consequently, inter-fiber nucleosome association can prevent the formation of 30-nm fibers by sequestering the H4 tail domain (Hansen 2012).

## Presence of 30-nm chromatin fibers in specific cells

Although inter-fiber nucleosome associations supposedly dominate within cells, there are some specific cell types whose nuclei contain apparent 30-nm chromatin fibers, for example chicken erythrocytes (Langmore and Schutt 1980; Woodcock 1994; Scheffer et al. 2011) and starfish sperm (Woodcock 1994; Scheffer et al. 2012). These cells are terminally differentiated, and so, transcription is almost silenced. In mouse rod cells, a large dense heterochromatin domain is located in the center of the nucleus. The heterochromatin at the periphery of the domain is formed by closely packed 30-nm fibers, whereas such fibers have not been detected in the centermost domain (Kizilyaprak et al. 2010). We propose that the stable formation of 30-nm chromatin fibers in these cells could play a role in robust gene silencing. Nevertheless, there must be a unique mechanism to facilitate intra-fiber nucleosome association in these specific cells. One possibility is the presence of a larger number of linker histones. Consistent with this, linker histones could stabilize 30-nm chromatin fibers in vitro (for a review, see Hansen 2002). In chicken erythrocytes, linker histone H5 is deposited in the chromatin fibers at ∼1.4 molecules/nucleosome (for a review, see Kowalski and Palyga 2011), whereas starfish sperm chromatin has ∼1.7 H1 molecules per nucleosome; in contrast, various somatic cells have 0.5−0.8 H1 per nucleosome (Woodcock et al. 2006). Specific histone modifications or the binding of specific proteins might also be involved in the formation of stable 30-nm fibers for robust gene silencing (Kowalski and Palyga 2011). Interestingly, the 30-nm fibers of peripheral heterochromatin in mouse rod photoreceptor cells contain acetylated histones, which are usually associated with active transcription and de-condensed (Kizilyaprak et al. 2010). Histone acetylation might induce the isolation of nucleosome fibers (Fig. 4a) and subsequent intra-fiber nucleosomal association to form stable 30-nm chromatin fibers because histone acetylation seems to inhibit inter-fiber nucleosome association by repelling the increasingly negative charge of the nucleosomes (Szerlong et al. 2010; Liu et al. 2011).

## Higher order interphase chromatin structures

**Fig. 5:**
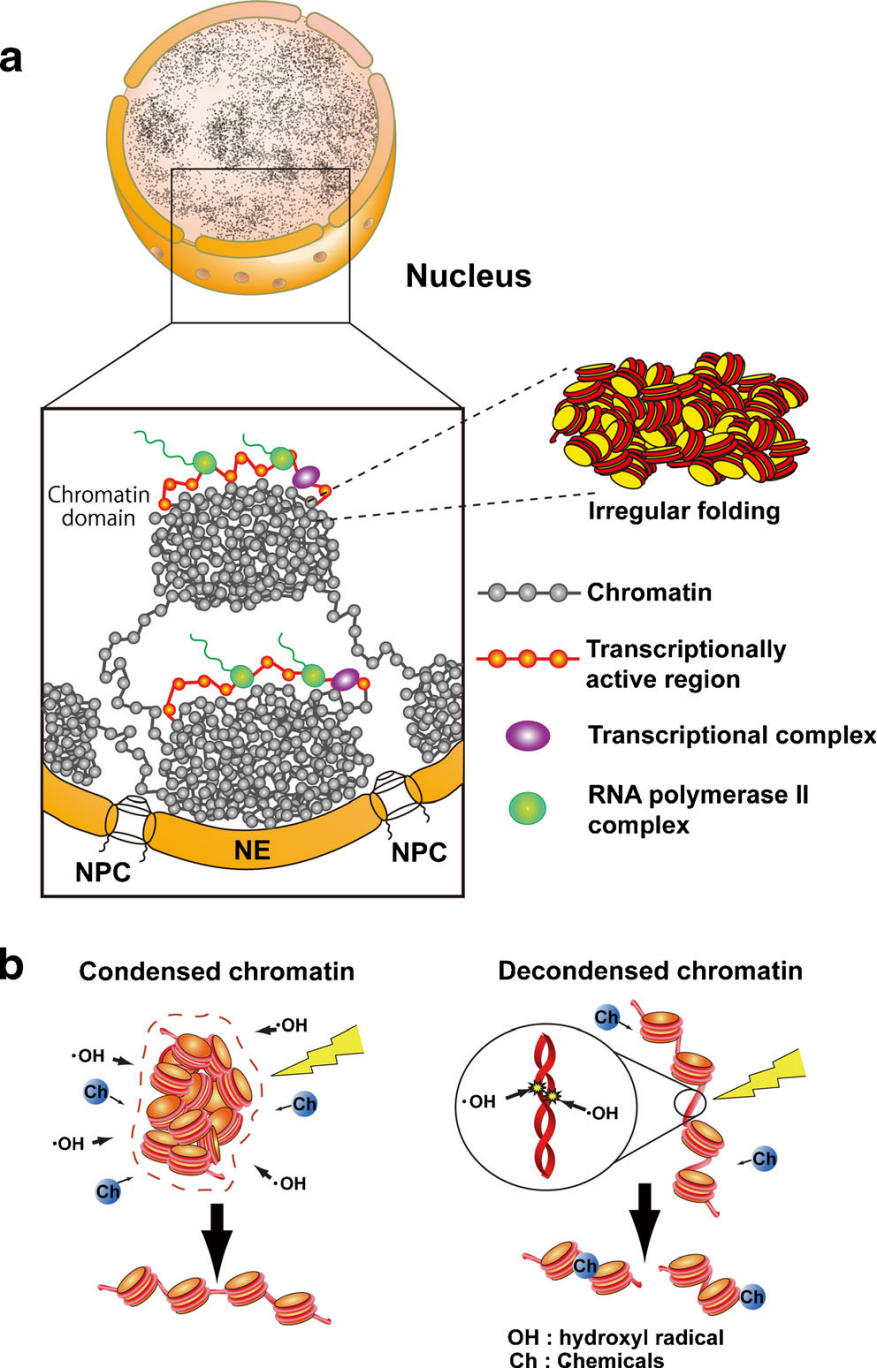
Higher order structure of interphase chromatin. **a** Condensed chromatin domains. Active chromatin regions are transcribed on the surfaces of chromatin domains with transcriptional complexes (*purple spheres*) and RNA polymerase II (*green spheres*). *NPC* nuclear pore complex, *NE* nuclear envelope. **b** (*Left*) Condensed chromatin is more resistant to radiation damage or chemical attack. *(Right*) Reactive radicals arising from the radiolysis of water molecules by irradiation can damage decondensed chromatin; decondensed chromatin is also more accessible to chemicals (labeled “Ch”)

As described above, several studies have demonstrated that irregular folded 10-nm nucleosome fibers form the bulk structure of interphase chromatin and mitotic chromosomes. Nevertheless, the higher order structure of chromatin must also be considered. Based on the available data, including studies from our laboratory, we propose that interphase chromatin forms numerous condensed chromatin domains consisting of irregularly folded 10-nm nucleosome fibers that resemble “chromatin liquid drops” (Fig. 5a) (Maeshima et al. 2010a; Joti et al. 2012). These domains can be considered to be drops of viscous chromatin, which could be formed by the macromolecular crowding effect (Asakura and Oosawa 1954) and other specific proteins such as cohesin (Nasmyth and Haering 2005; Hirano 2006) and/or condensin II (Ono et al. 2013; Thadani et al. 2012). Similar chromatin domains were proposed in the chromosome territory-interchromatin compartment (CT-IC) model (Cremer et al. 2000; Cremer and Cremer 2001), where each CT is built from a series of interconnected 1 Mb-sized chromatin domains. These domains were identified originally using pulse labeling of DNA replication foci (Nakamura et al. 1986; Schermelleh et al. 2001; Berezney et al. 2005; Albiez et al. 2006) that persisted stably in subsequent cell generations (Jackson and Pombo 1998; Ma et al. 1998; Zink et al. 1999). Several recent reports have used the Hi-C and chromosome conformation capture carbon copy (5C) methods to investigate the three-dimensional architecture of genomic DNA within cells, and they have proposed the physical packaging of genomic DNA. The DNA packing units were termed “topologically associating domains (TADs)” (Nora et al. 2012), “topological domains” (Dixon et al. 2012), or “physical domains” (Sexton et al. 2012). Recent studies have reported that TADs, which can be hundreds of kilobases in size, were identified in fly, mouse, and human cells, suggesting that TADs could be universal building blocks of chromosomes. Loci located within TADs tend to interact frequently with each other, but they interact much less frequently with loci located outside their domain.

What is the advantage of these condensed chromatin domains? A number of biological implications have been proposed for TADs (Nora et al. 2013). For instance, TADs were found to correspond to lamin-associated chromatin domains (LADs) in nuclei (Guelen et al. 2008). Most DNA replication domains, where DNA replication takes place in a nearly synchronous manner, overlap with multiple TADs (Ryba et al. 2010). Changes in timing during cell differentiation typically involve TAD-sized regions. Regarding transcriptional regulation, enhancer-promoter interactions produced by looping might be limited to elements located within the same TAD (Shen et al. 2012). TADs might also be defined by genetically encoded boundary elements (Nora et al. 2012).

In addition, we reported recently that condensed chromatin is more resistant to radiation damage than the decondensed form (Fig. 5b), presumably because condensed chromatin has a lower level of reactive radical generation after ionizing irradiation (Takata et al. 2013). The condensed state also protects genomic DNA from chemical attack. These findings suggest that condensed chromatin domains play an important role in maintaining genomic integrity (see also Falk et al. 2008).

## Mitotic chromosome structure

**Fig. 6:**
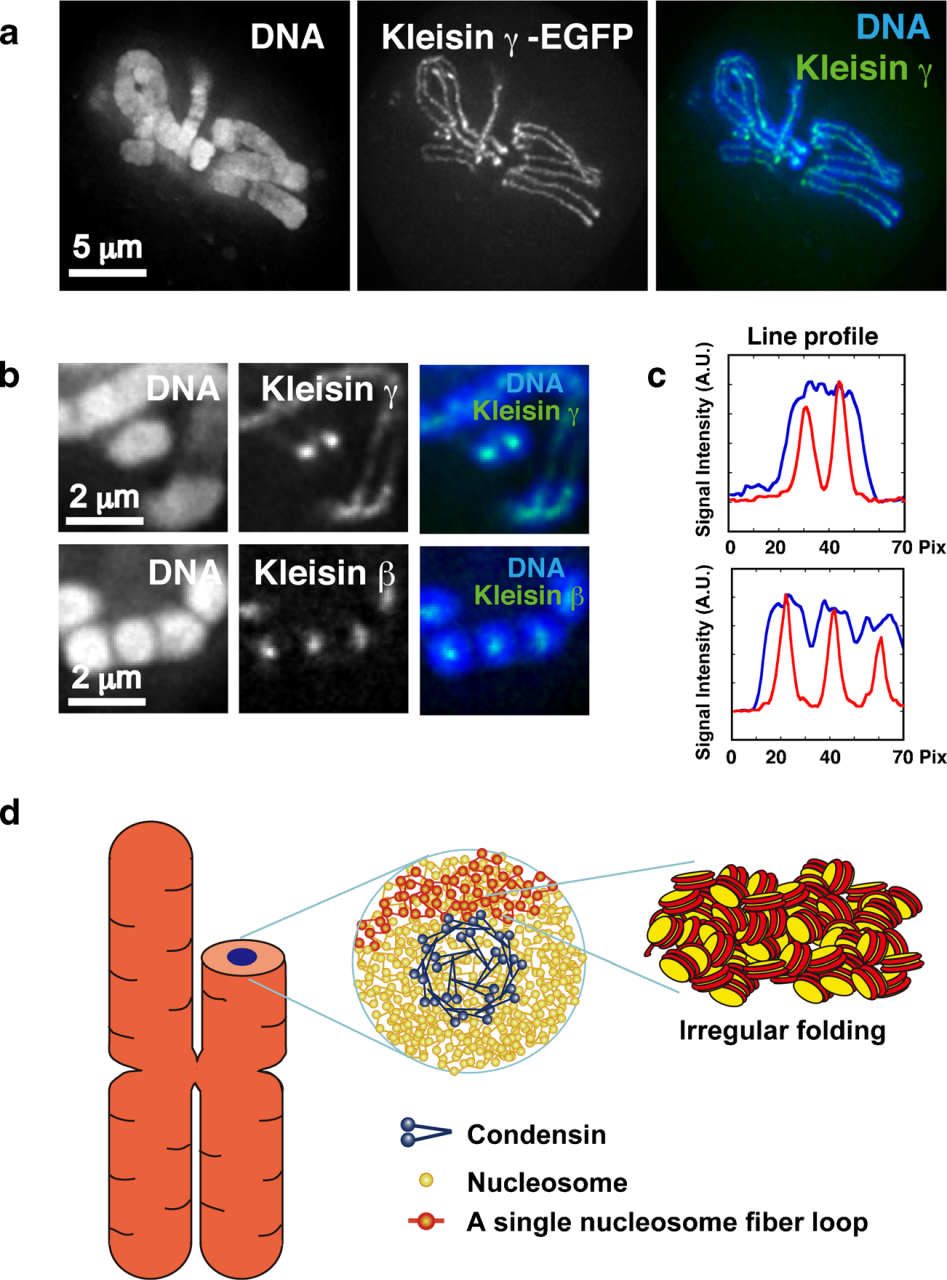
Mitotic chromosome structure. Axial localizations of condensins I and II in mitotic chromosomes in live mammalian cells. For DNA staining, DM (Indian Muntjac cells) cells stably expressing EGFP-Kleisin γ (condensin I) and EGFP-Kleisin β (condensing II) were stained with Hoechst 33342. Live-cell imaging was performed using a Delta Vision microscope (applied precision). **a** Clear axial signals of EGFP-Kleisin γ in mitotic chromosomes are shown. **b** End-on-view of mitotic chromosomes. The *upper panel* shows DM cells expressing EGFP-Kleisin γ, whereas the *lower panel* shows DM cells expressing EGFP-Kleisin β stably. Restricted dot signals from two types of EGFP-Kleisin in the cross-section of a chromosome body (DNA staining) are shown. **c** Quantitative data using line-profile analysis (*blue line*, DNA; *red line*, Kleisin signals) is shown. There is clear axial localization of condensins I and II in mitotic chromosomes in live mammalian cells. **d** Chromosomes consist of irregularly folded 10-nm nucleosome fibers. Condensins (*blue*) hold the nucleosome fibers (*red*) around the center of the chromosome. Locally, the nucleosome fibers are folded in an irregular or disordered manner, forming loop structures that collapse towards the center of the chromosome center (*blue*). The collapsed fiber (*red*) then forms a domain

Nucleosome fibers (10-nm) are somehow organized into mitotic chromosomes. Condensins and topoisomerase IIα, which are essential for chromosome condensation, form an axis in the chromosome in various cell types (Hirano 2012; Thadani et al. 2012; Ohta et al. 2010; Belmont 2006; Maeshima and Eltsov 2008). Although it was claimed that the condensin axis was observed only in fixed and not living cells (Thadani et al. 2012), we observed clear axial structures of the condensin structures kleisin β- and γ-EGFP in chromosomes in living mammalian cells (Fig. 6a–c). Therefore, we hypothesized that condensins hold 10-nm nucleosome fibers around the chromosome center creating loops, as proposed in the radial loop/scaffold model (Figs. 2c and 6d) (Laemmli et al. 1978; Maeshima and Eltsov 2008; Nishino et al. 2012). Locally, nucleosome fibers are folded in an irregular manner towards the center of the chromosome (Fig. 6d) (Nishino et al. 2012). An immuno-EM study of condensins revealed a traceable condensin array near the center of chromosome cross-sections (Maeshima et al. 2005), suggesting the oligomerization or self-assembly structure of condensins, which capture nucleosome fibers. Condensins can also aggregate in the presence of DNA (Yoshimura et al. 2002; see also Hirano 2012). In our model, the orientation of nucleosome fibers in chromosomes is isotropic (Fig. 6d). This suggests that a specific locus of the genome is randomly incorporated into a wide ranging, but not reproducibly specific, region of the chromosome (Nishino et al. 2012). This is consistent with data reported using fluorescent labeling of specific chromosomal sites (Strukov and Belmont 2009).

Naumova et al. (2013) recently performed 5C and Hi-C analyses to understand the three-dimensional folding of genomic DNA in mitotic chromosomes. In human cells from G1 to S to G2 phase, they identified large chromatin structures called “chromosome compartments” (multi-megabases) and TADs (hundreds of kilobases), both of which were found in previous studies (Lieberman-Aiden et al. 2009; Nora et al. 2012; Dixon et al. 2012; Sexton et al. 2012). However, these structures were not found during mitosis; instead, they found homogenous folding of genomic DNA, which seems to be consistent with our view that chromosomes consist of irregularly folded nucleosome fibers.

Using polymer simulations, they found that the obtained data for mitotic chromosomes are inconsistent with the classic hierarchical helical folding model (Fig. 2c) and are, instead, best described by a linearly organized longitudinally compressed array of consecutive chromatin loops (Naumova et al. 2013), which is essentially similar to the radial loop/scaffold model (Fig. 2c) (Laemmli et al. 1978; for a review, see Kleckner et al. 2013).

## Dynamic 10-nm fibers in living mammalian cells

The original liquid chromatin model proposed by Dubochet (McDowall et al. 1986; Dubochet et al. 1988) and our polymer melt model (Eltsov et al. 2008; Maeshima et al. 2010a) both imply a less physically constrained chromatin state and a more locally dynamic state; the 10-nm nucleosome fibers fluctuate locally. Therefore, we attempted to visualize local nucleosome fluctuation. Previous studies of chromatin dynamics employed very large chromatin regions such as the LacO array that encompasses 20−50 nucleosomes (Straight et al. 1996; Belmont et al. 1999; Heun et al. 2001; Vazquez et al. 2001; Chubb et al. 2002; Levi et al. 2005; Hajjoul et al. 2013). The motion of these large regions in living mammalian cells was measured by monitoring the movement of the GFP-LacI signal bound to the LacO array at specific chromatin regions.

**Fig. 7:**
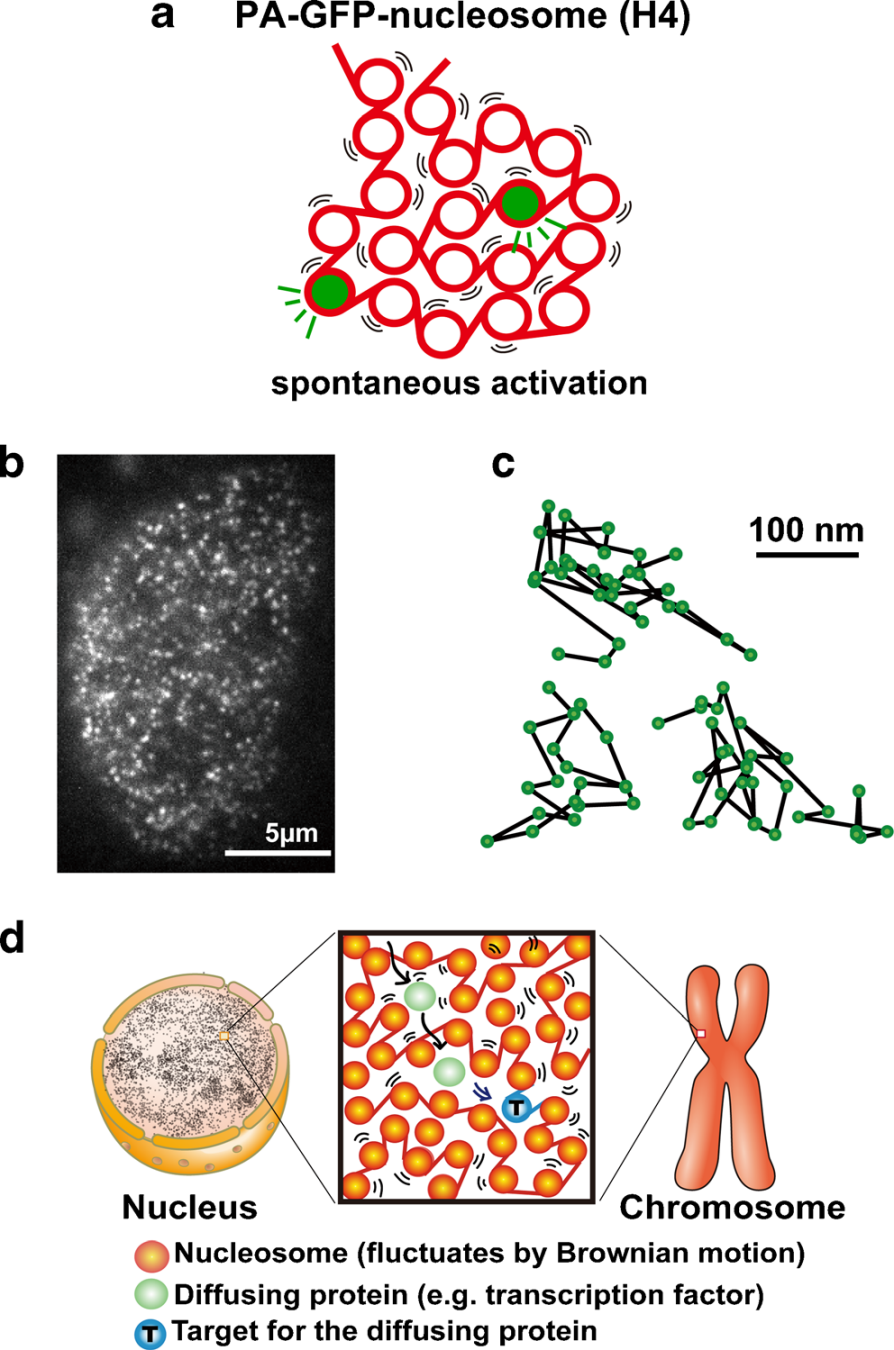
Single nucleosome imaging. **a** A small portion of PA-GFP-H4 was activated spontaneously without laser activation and was used for single nucleosome imaging. **b** Single nucleosome image of a DM cell (Indian Muntjac cell) nucleus that expresses PA-GFP-H4. PA-GFP-H4 is observed as a bright dot using oblique illumination microscopy. The dots were fitted to an assumed Gaussian point spread function to determine the precise center of signals with higher resolution. Bar = 5 μm. **c** Representative three trajectories of fluorescently tagged single nucleosomes. **d** Chromatin fluctuations as a basis for scanning genome information. In cells, nucleosome fibers (*red spheres and lines*) are folded irregularly. The nucleosomes fluctuate, and these nucleosome dynamics facilitate chromatin accessibility. The images were reproduced from (Hihara et al. 2012; Nozaki et al. 2013) with some modification

To observe and analyze more local nucleosome dynamics, we performed single nucleosome imaging in living mammalian cells (Fig. 7) (Hihara et al. 2012; Nozaki et al. 2013). We fused histone H4 with photoactivatable (PA)-GFP and expressed the fusion protein in mammalian cells at a very low level (Fig. 7a). We then used an oblique illumination microscope to illuminate a limited thin area within the cell for single nucleosome imaging (Hihara et al. 2012; Nozaki et al. 2013; for principle, see Tokunaga et al. 2008). Generally, PA-GFP shows green fluorescence only after activation with a 405-nm laser (Lippincott-Schwartz and Patterson 2009). Surprisingly, we observed that a small fraction of H4-PA-GFP and PA-GFP-H4 in the cells was activated spontaneously without laser stimulation (Fig. 7a). Figure 7b shows a typical single nucleosome image of a living mammalian cell. Each bright dot in the nucleus represents a single H4-PA-GFP (PA-GFP-H4) within the single nucleosome. Strikingly, we observed significant nucleosome fluctuation (∼50 nm movement/30 ms) in both interphase chromatin and mitotic chromosomes (Fig. 7c), which is likely caused by the Brownian motion (Hihara et al. 2012; Nozaki et al. 2013). Mean square displacement (MSD) plots, measuring the spatial extent of the random motion, and fitting to an anomalous diffusion curve suggested a restricted nucleosome movement. The McNally group also published single nucleosome tracking data using H2B-EGFP (Mazza et al. 2012), which appears to be consistent with our single nucleosome tracking results using PA-GFP-H4.

## Local fluctuation of nucleosomes as a basis for scanning genome information

Some computational modeling studies, including our own, have suggested that nucleosome fluctuations facilitate the mobility of diffusing proteins in the chromatin environment (Fig. 7d) (Hihara et al. 2012; see also Wedemeier et al. 2009; Fritsch and Langowski 2011). Such nucleosome fluctuations may also contribute to the frequent exposure of genomic DNA sequences. Because both facilitating protein mobility and DNA exposure increase chromatin accessibility, these local dynamics may be advantageous in template-directed biological processes such as transcriptional regulation, DNA replication, and DNA repair/recombination. Therefore, we propose that the local fluctuation of nucleosomes forms the basis for scanning genome information (Fig. 7d).

We consider that nucleosome fluctuations are involved in various cellular functions. Hinde et al. (2012) examined the chromatin dynamics in human ES cells based on signal intensity fluctuations of DAPI or H2B-EGFP, and they found that the intensity of the fluctuations in ES cells was drastically impaired during differentiation, suggesting that such fluctuations correlate with pluripotency. A dynamic chromatin state may be required for high transcriptional competency to maintain pluripotency. Elucidation of the spatio-temporal regulation of nucleosome fluctuations would be an intriguing next step.

## Conclusions

The traditional view of chromatin is changing from one of static regular structures including 30-nm chromatin fibers to a dynamic irregular folding structure of 10-nm nucleosome fibers. Although the term “irregular” or “disordered” might give the impression that the organization is functionally irrelevant, the irregular folding results in less physical constraint and increased dynamism, increasing the accessibility of the DNA (Fig. 7d). This dynamic state may be essential for various genome functions, including transcription, replication, and DNA repair/recombination.

A new paper (Eltsov M, Sosnovski S, Olins AL, Olins DE: Chromosoma. 2014 Feb 26. [Epub ahead of print]) published after this article went to press. The authors studied nuclear envelope-limited chromatin sheets (ELCS) by cryo-EM. They found that the 30-nm chromatin fibers could only be observed following aldehyde fixation; none were seen in cryo-sections, suggesting that the 30-nm chromatin fibers in ELCS visualized by conventional EM could be an artifact structure.
